# Probiotics in Autoimmune and Inflammatory Disorders

**DOI:** 10.3390/nu10101537

**Published:** 2018-10-18

**Authors:** Yuying Liu, Jane J. Alookaran, J. Marc Rhoads

**Affiliations:** The Department of Pediatrics, Division of Gastroenterology, The University of Texas Health Science Center at Houston McGovern Medical School, Houston, TX 77030, USA; Yuying.Liu@uth.tmc.edu (Y.L.); Jane.J.Alookaran@uth.tmc.edu (J.J.A.)

**Keywords:** lactobacilli, bifidobacilli, arthritis, inflammatory bowel, microbiome, metabolomics, aryl hydrocarbon reductase, adenosine, histamine, short-chain fatty acid

## Abstract

Probiotics have been used to ameliorate gastrointestinal symptoms since ancient times. Over the past 40 years, probiotics have been shown to impact the immune system, both in vivo and in vitro. This interaction is linked to gut microbes, their polysaccharide antigens, and key metabolites produced by these bacteria. At least four metabolic pathways have been implicated in mechanistic studies of probiotics, based on mechanistic studies in animal models. Microbial–immune system crosstalk has been linked to: short-chain fatty acid production and signaling, tryptophan metabolism and the activation of aryl hydrocarbon receptors, nucleoside signaling in the gut, and activation of the intestinal histamine-2 receptor. Several randomized controlled trials have now shown that microbial modification by probiotics may improve gastrointestinal symptoms and multiorgan inflammation in rheumatoid arthritis, ulcerative colitis, and multiple sclerosis. Future work will need to carefully assess safety issues, selection of optimal strains and combinations, and attempts to prolong the duration of colonization of beneficial microbes.

## 1. History of Probiotics

Health benefits of bacteria have been recognized throughout history. Fermented milk was consumed in the Middle East as early as 10,000 BC, followed by populations in Egypt (as evidenced by hieroglyphics), Greece, and Italy [1]. Around 8000 BC, Tibetan nomads living at altitudes >4000 m maintained good health despite the absence of fruits and vegetables in their diet, in part by consuming fermented yak milk and its products [2]. Only eight ounces of yak milk daily could provide >200 billion lactobacilli, mainly *Lactobacillus fermentum* (*L. fermentum*) and *L. casei*. Yak milk also has been found to have free-radical-scavenging and anti-inflammatory properties. In ancient Greece and Rome around 400 BC, a condiment called *garum*, derived from fish intestines, which was (and still is) fermented for 12–18 months in clay pots, was consumed daily, with powerful antioxidant properties and reported health benefits [3]. Nomadic Turks used “yogurmak” to treat diarrhea, cramps, and sunburned skin, as evidenced by writings in the 11th century; and later, Genghis Khan, the great Mogul conqueror, fed his army yogurt, because it reportedly “instilled bravery in them” [4].

In 1905, Elie Metchnikoff of Russia probed the question of why Bulgarians lived so long. He concluded that their longevity was related to the heavy consumption of fermented yogurt, subsequently showing that a bacillus could be grown from the yogurt, which was identical to a bacillus found in their stools, later called *L. bulgaricus* [5]. At the same time, Henry Tissler of Paris isolated from an infant a y-shaped organism that he called *Bifidobacterium.* This bacterium was able to displace pathogenic bacteria in vitro. Healthy infants were colonized with the *Bifidobacterium*, whereas less healthy infants did not harbor the organism. Later, in World War I, many soldiers were dying of diarrheal disease, and the German scientist Alfred Nissle isolated a strain of *E. coli* from a soldier that had *Shigella* in the stool but did not develop diarrhea [6]. The species, which he called “antagonistically strong”, was appropriately called *E. coli* Nissle 1917 and is still used as a probiotic today (called “Mutaflor”).

### 1.1. Recognized Benefits in the 1900s

The term probiotic was introduced in 1953 by the German Werner Kollath to mean “active substances essential for a healthy life” [5]. In the 1940s, most research focused on culturing pathogenic bacteria and developing antimicrobial therapies. In line with this approach, after the 1950s, there was great interest in identifying probiotics that provided colonization resistance to pathogens, and research began to focus on lactobacilli and bifidobacilli to combat diarrheal disease. This research focused on the role of probiotics and “gut health” and resulted in convincing evidence that probiotics can prevent and treat infections causing diarrhea (viral, salmonellosis, shigellosis, cholera) [7] and also facilitate peptic ulcer healing [1].

### 1.2. Expanded Role in Infants and in Patients with Gastroinestinal Disorders (2000–Present)

Between 2000 and 2017, there was an explosion of interest in probiotics, with an annual number of randomized controlled trials (RCTs) ranging between 144 and 194; in 2017, there were also 49 meta-analyses. Internationally recognized investigators have spent decades of their lives developing the field of probiotic research.

Four significant conditions will be mentioned that have consistently been shown to respond to probiotics in humans in meta-analysis.

*Necrotizing enterocolitis (NEC)*. Research was emerging around 2000 showing that probiotics could prevent necrotizing enterocolitis, a devastating disease of premature infants often resulting in bowel resection and short bowel syndrome. The first meta-analysis by Alfaleh and Bassler was published in 2008, showing benefit of probiotics in nine trials [8]. By 2017, more than 23 studies in 7325 infants showed that probiotics reduce the risk of developing NEC. This most recent meta-analysis by Thomas et al. showed that the risk of developing NEC was 3.9% if given probiotics and 6.6% if untreated with probiotics (relative risk of 0.57, 95% confidence interval (CI) 0.43–0.74, *p* < 0.0001) [9]. The problem with these studies was that there were many probiotics studied, and sometimes multiple-strain probiotics were tested; therefore, the optimal choice was not evident. One meta-analysis found that the benefit was restricted to multiple-strain probiotics and to lactobacilli [10], while another meta-analysis (oppositely) found that the benefit pertained only to bifidobacilli and multiple-strain probiotics [11]. Both groups found that the yeast *Saccharomyces* was ineffective.

Of concern, the premature population is at high risk for septicemia, and therefore safety concerns have until recently led to the U.S. Food and Drug Administration (FDA)’s caution in approving any probiotic RCTs in the United States. Paradoxically, probiotics have been consistently shown to reduce the risk of late-onset septicemia in breast-fed premature infants [12]. Simultaneously with these clinical trials, animal research has confirmed efficacy of probiotics in preventing NEC, while also establishing possible mechanisms. Dvorak’s group showed that *Bifidobacterium bifidum* stabilized the gut barrier via tight junction modification during experimental NEC [13]. Hackam’s group showed that NEC is mediated by inflammatory signaling via the epithelial cell pattern recognition receptor Toll-like receptor-4 (TLR4). TLR4 recognizes bacterial lipopolysaccharide and is expressed on gut epithelial cells and immune cells, such as T cells. Hackam et al. showed that the mitigating effects of *L. rhamnosus* HN001 are mediated by anti-inflammatory signaling via TLR9 [14]. Our group showed that protective effects of the probiotic *Lactobacillus reuteri* DSM 17938 (*L. reuteri* 17938) in a mouse model of NEC are mediated by a different Toll-like receptor, TLR2 [15], and its administration to newborn mice and rat pups results in an enhancement of local and peripheral levels of anti-inflammatory regulatory T cells (Tregs) [16].

*Irritable bowel syndrome (IBS)*. IBS is defined as recurrent abdominal pain at least one day weekly for >3 months, which is: (a) related to defecation; (b) associated with a change in stool form; or (c) related to a change in stool [17]. Subjects with IBS have been found to harbor an altered fecal microbial population, with a shift toward reduced microbial diversity and reduced butyrate-producing bacteria. In addition, Pozuelo et al. showed that adults with IBS-C (constipation-predominant) differ from control individuals without IBS and from those with IBS-D (diarrhea-predominant IBS) [18]. This finding was consistent with many studies of probiotics for patients with IBS. Meta-analyses have shown considerable heterogeneity, largely related to various definitions of symptom severity in IBS and quality-of-life indicators. However, most meta-analyses have shown efficacy of probiotics in treating IBS [19]. The most recent meta-analyses by Ford et al. [19] and Zhang et al. [20] showed a decrease in global IBS symptoms of ~2-fold and an improvement in quality of life. Many studies showed improvement in bloating and flatulence in those with IBS. Different probiotics have been studied, and the meta-analyses have shown considerable heterogeneity. Therefore, the role of probiotics in IBS is best described as “evolving but promising.”

*Infant colic*. Babies who cry and fuss for more than 3 h daily have colic. The condition generally starts at 3 weeks of age, occurs on more than 3 days/week, and resolves after 3 months of age (hence the “rule of threes” [21]. Infant colic previously was felt to be unresponsive to any treatment. Microbial dysbiosis began to be linked to this condition and was confirmed by several groups [22,23,24], and it was linked to gut inflammation [25]. Therefore, colic might represent a condition for which probiotic treatment would be useful. Several meta-analyses have shown that the probiotic *L. reuteri*, isolated from a Peruvian mother’s breast milk, reduces crying time and irritability in this condition [26,27,28].

*Respiratory infections.* Recently, lactobacillus- and bifidobacillus-containing probiotics were found to improve outcomes in acute infectious diseases outside of the gastrointestinal tract, such as upper and lower respiratory tract illnesses in infants and college students [29,30,31,32]. In one moderately large multicenter study in Italy, the addition of fermenting *L. paracasei* to milk or rice milk resulted in reduced episodes of gastroenteritis, rhinitis, otitis, laryngitis, and tracheitis [33]. This finding suggested that the benefits to the host extend beyond local interactions in the intestinal tract between the gut organisms, enterocytes, and the immune system, perhaps involving microbial metabolites and/or migrating dendritic cells that reach distant locations such as the spleen and lymph nodes. Of additional benefit, probiotics stimulate immunoglobulin A (IgA) secretion in the respiratory epithelium in animal models [34]. Currently, several over-the-counter products espouse the benefits of probiotics in treating common upper respiratory ailments.

## 2. Effects of Probiotics in High-Risk Populations with Immune Dysregulation and Autoimmune Diseases

In both animal trials and human trials, probiotics have been investigated to determine potential beneficial effects in the prevention and treatment of a wide variety of systemic conditions. These conditions include inflammatory and autoimmune diseases such as rheumatoid arthritis, ulcerative colitis, multiple sclerosis, and hepatic encephalopathy. Advantages of probiotics include the regulation of immune system function, which is often dependent on the strain of probiotic bacteria. Some strains have demonstrated stimulation of the immune response, thereby being beneficial to patients suffering from immunodeficiency [35]. Other strains have been shown to inhibit the immune response, thereby being beneficial for patients suffering from conditions with immune activation such as rheumatoid arthritis (RA) [36,37].

*Rheumatoid arthritis (RA)*. Rheumatoid arthritis is a systemic autoimmune disease characterized by autoantibody formation leading to the chronic inflammation of multiple joints. RA is also known to affect other internal organs, including the lungs, heart, and kidneys [38]. Triggers leading to RA include human leukocyte antigen (HLA) gene interaction and environmental factors. These environmental factors include smoking, infection, and recently, dysbiosis [36,39,40]. Early animal models have consistently demonstrated an interaction between the gut microbiota and local/systemic immunity as well as activation of joint inflammation [41]. In earlier probiotic studies, investigators were not able to show a significant difference in activity of RA with the use of probiotics [42], but in a more recent study, Zamani et al. reported that probiotic supplementation resulted in improved disease activity scores (looking at 28 joints) in patients with RA, compared with placebo [43]. A study by Chen et al. evaluated the gut microbiota profile in 40 patients with RA and 32 healthy controls. They found decreased gut microbial diversity in RA compared to controls, which additionally correlated with disease duration and with levels of serum rheumatoid factor [44]. Alipour et al. showed that *L. casei* 01 supplementation decreased serum high-sensitivity C-reactive protein (hs-CRP) levels, reduced tender and swollen joint counts, and improved global health (GH) score (*p* < 0.05). A significant difference was also observed between the two groups with respect to circulating levels of interleukin (IL)-10, IL-12, and tumor necrosis factor (TNF)-α, in favor of the probiotic group [45].

In a recent meta-analysis, Mohammed et al. showed that the proinflammatory cytokine IL-6 was significantly lower in rheumatoid arthritis volunteers treated with probiotics compared to their placebo-treated controls. However, this study did not show an overall difference in clinical symptoms between the probiotic and placebo groups [37]. Another study, by Liu et al., aimed to investigate the human fecal lactobacillus community and its relationship to RA. In comparing quantitative polymerase chain reaction (PCR) in fecal samples of 15 RA patients and 15 healthy controls, the authors reported increased absolute numbers of *Lactobacillus salivarius*, *Lactobacillus iners*, and *Lactobacillus reminis* in untreated RA patients and suggested a potential relationship between the lactobacillus community and development of RA [46]. Thus, evolving evidence suggests a relationship between altered intestinal microbiota and rheumatoid arthritis, and we anticipate that further studies will be needed to delineate the microbiota profiles which might contribute to RA and the potential for treatment with adjuvant probiotics.

*Systemic lupus erythematosus (SLE)*. SLE is an autoimmune disease involving multiple organs, including the skin, joints, kidneys, and central nervous system and is characterized by the formation of high levels of antibodies against double-stranded DNA. SLE is influenced by genetic and environmental factors and is characterized by immune intolerance to self-antigens [47]. In a classic hypothesis regarding the etiology of lupus in 1964, Kingsley Stevens pointed out that polysaccharide-containing antigens were 60-fold more effective stimulators of plasma cell proliferation and antibody formation than were the protein antigens present in vaccines [48]. He went on to propose that “the causative agent in SLE” is a bacterial polysaccharide, which must be present in the oropharynx, vagina, or gut. In humans with SLE, elevated interferon-gamma has been found to be proportional to the fecal firmicutes/bacteroides level, giving credence to Stevens’ hypothesis [49]. In this study, several strains of probiotics were helpful in the modulation of excessive inflammatory responses in vitro. Both experimental and clinical trials have revealed that selective strains of probiotics (*B.* bifidum, *Ruminococcus obeum*, *Blautia coccoides*, and *L. casei* strain Shirota) can reduce inflammation and restore tolerance in SLE animal models [50]. There are several mouse models of SLE; for example, the MRL/lpr mouse that spontaneously develops nephritis. MRL/lpr mice suffer from endodoxemia and increased gut paracellular permeability [51]. Using MRL/lpr mice, researchers found that combinations of lactobacilli or *L. reuteri* alone, when given enterally, skewed Treg–Th17 balance toward Treg cell dominance, reduced endotoxemia, reduced levels of double-stranded DNA-reactive IgG, improved proteinuria, and better survival. These results were associated with a change in gut microbiota, with expansion of Clostridiales, Lactobacilli, and Desulfovibrionales. In the NZB/W F1 mouse, systemic lupus-like inflammation is characterized by oxidative stress and reduced levels of circulating regulatory (anti-inflammatory) Tregs [52]. Treatment with *L. reuteri* GMNL-263 reduced levels of cytokines and restored Tregs in this model as well.

At this time, we are unaware of any randomized controlled trials of a probiotic for patients with lupus, but there is evidence that the gastrointestinal tract may be an avenue for disease modification. In vitro, probiotic lactobacilli when cultured with immature dendritic cells from lupus patients reduce the expression of costimulatory molecules and increase levels of interleukin-10 and indoleamine 2,3-dioxygenase (anti-inflammatory molecules), suggesting that they could promote immune tolerance [53]. A pilot study by Frech et al. in a related autoimmune disorder, progressive systemic sclerosis, suggested that probiotics significantly improved esophageal reflux, distention and bloating, and total gastrointestinal symptom scales [54].

*Inflammatory bowel disease (IBD)*. IBD, including ulcerative colitis (UC) and Crohn’s disease (CD), is characterized by chronic inflammation in the gastrointestinal tract influenced by several factors, including genetics, epigenetics, gut microbiota, and the host immune system [55]. There have been many RCTs evaluating the effects of probiotics in IBD, associated with ample evidence suggesting that altered gut microbiota contribute to the initiation and progression of IBD. It has been well established that VSL #3, an eight-strain probiotic which includes lactobacilli, bifidobacilli, and *Streptococcus thermophilus*, is effective in UC; however, this and other probiotics were not effective in CD [56]. In 2017, Derwa et al. showed VSL #3 to be effective in inducing remission in active UC and suggested that probiotics may be as effective as 5-ASAs in preventing relapse of quiescent UC [57,58]. In a recent meta-analysis of 27 trials, Ganji-Arejanaki et al. confirmed that VSL #3 was effective in UC and showed that probiotics *S. boulardii*, Lactobacilli (*L. rhamnosus*, *L. johnsonii*), and VSL #3 were effective in patients with CD who also used corticosteroids [59]. The authors suggested that the use of VSL #3 and *Lactobacillus johnsonii* after surgery for CD might be efficacious if the duration of treatment under study were longer. Ganji-Arejanaki et al. additionally concluded that in children aged 2–21 years with IBD (both CD and UC), lactobacilli (*L. reuteri* ATCC 55730, *L. rhamnosus* strain GG, and VSL #3) confer a significant advantage. The role of probiotics in patients with persistent gastrointestinal complaints when inflammation cannot be demonstrated remains to be determined. Overall, in inflammatory bowel disease, probiotics appear to be safe and promising, but not proven as adjuvants to standard therapy [57].

*Multiple sclerosis (MS)*. MS is a chronic relapsing or progressive disease of the brain and spinal cord characterized by onset in early to middle adulthood with relapsing neurologic deterioration. Many individuals with MS develop sensory loss, weakness, visual difficulties, severe fatigue, and paresthesias. Key pathological features of MS include axonal loss, demyelination, gliosis, and a progressive inflammatory reaction of the brain and spinal cord [60,61]. During the course of MS, activated autoreactive T cells have been proposed to differentiate into interferon-γ-producing T helper 1 (T_H_1) cells and/or interleukin (IL)-17-producing T_H_17 cells, which are distributed throughout the central nervous system and spinal cord [62]. Growing evidence from both rodent and human studies suggests that microbiota within the intestine contribute to the pathogenesis in this disease [63,64,65,66]. In a rodent model of MS called experimental autoimmune encephalomyelitis (EAE), two studies showed that alteration of the gut microbiota by oral antibiotic administration reduced the severity of EAE [67,68]. Human studies of MS patients recently showed that the relative abundance of the families *Prevotella* and *Lactobacilli* are decreased compared to healthy controls [64,65]. Similarly, we found in the EAE model that there was evidence for fecal microbial dysbiosis and reduction of *Prevotella* during the disease. We also found that *L. reuteri* improved clinical severity of EAE, shifted the microbial beta diversity, and reduced Th1 and Th17 cytokine levels in the serum and gut [69]. There is one human study suggesting that *L. reuteri* improves symptoms and quality of life in human MS [70]. Thus, evidence from MS in humans and mice provides further evidence of a strong connection between the human brain and gut, with microbes and their products being key mediators of disease severity, while beneficial microbes represent key candidates for disease modification.

## 3. Mechanism of Action of Probiotics

**Figure 1:**
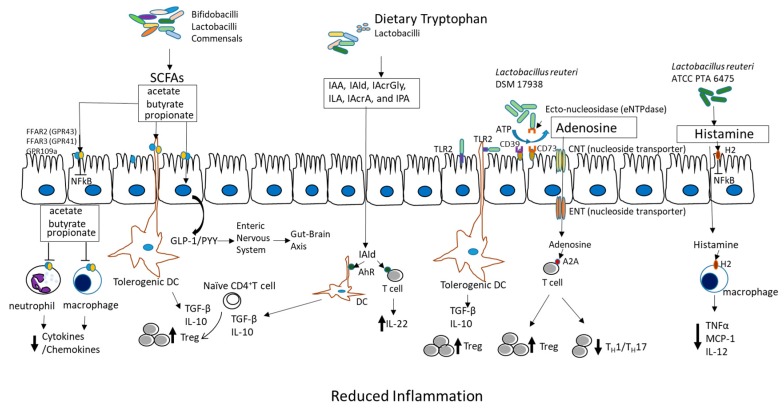
Critical metabolites produced by probiotics which have anti-inflammatory functions. SCFAs (acetate, butyrate, and propionate) produced by bifidobacilli, lactobacilli, and commensals bind and activate receptors (FFAR2, FFAR3, or GPR109a) on intestinal epithelial cells to inhibit the nuclear factor kappa-light-chain-enhancer of activated B cells (NF-κB) pathway to prevent inflammation. They also inhibit histone deacetylases to promote accumulation of Tregs and may release GLP1/PYY to act on the enteric nervous system and the CNS to affect energy homeostasis and gut motility. SCFAs also induce tolerogenic DC, which educate naïve CD4^+^ T cells to differentiate into Tregs. These actions inhibit cytokine production by neutrophils and macrophages via interaction with receptors. Dietary tryptophan and probiotic-produced indole derivatives interact with AhR expressed on immune cells to produce anti-inflammatory effects. *L. reuteri* 17938 promotes adenosine generation, most likely by an ectonuclease present on the probiotic itself and on intestinal epithelial cells. Adenosine and its derivative inosine interact with adenosine receptor-2A located on T cells to promote Treg functions and inhibit inflammatory T_H_1 and T_H_17 subsets. Histamine produced by *L. reuteri* 6475 interacts with H_2_ presented on intestinal epithelial cells and macrophages to reduce levels of proinflammatory cytokines (TNF-α, MCP-1, and IL-12). In summary, the critical metabolites produced by probiotics generate anti-inflammatory effects during diseases. Abbreviations: SCFAs: short-chain fatty acids; FFARs: free fatty acid receptors; GPRs: G-binding protein receptors; NF-κB: nuclear factor kappa-light-chain-enhancer of activated B cells; GLP1: glucagon-like protein-1; PYY: peptide tyrosine tyrosine; CNS: central nervous system; AhR: aryl hydrocarbon receptor; T_H_1 and T_H_17: T helper cells; H_2_: histamine receptor 2; TNF-α: tumor necrosis factor alpha; MCP-1: monocyte chemoattractant protein-1; IL-12: interleukin-12 (illustration by Yuying Liu).

Probiotics have been found to affect every compartment of the gut, including the luminal microbiome, the mucus barrier, the microbe- and cell-free “kill zone” of the epithelium, the lymphocyte- and plasma cell-rich lamina propria, the vascular and neural elements of the lamina propria, the underlying smooth muscles which control motility, and the mesenteric lymph nodes that communicate with the systemic immune system. Probiotic-modulated local and systemic metabolites have been identified which may modify autoimmune diseases and the mechanisms are summarized in Figure 1.

*Short-chain fatty acid (SCFA) production in the colon*. SCFAs, specifically acetate, propionate, and butyrate, are produced by commensal bacteria (such as *Facecalibacterium prausnitizii*, *Eubacterium rectale*, *Eubacterium hallii*, and *Ruminococcus bromii*) and by many probiotics. Lactobacilli were once thought to produce SCFAs and pyruvate by fermentation of carbohydrates and heterofermentative processes [71]; however, they do not produce SCFAs directly. As members of a trophic chain leading to butyrate production, they produce mainly lactate [72]. Lactate is further metabolized by strictly anaerobic butyrate producers from the Firmicutes phylum (which includes *Lachnospiraceae*, *Ruminococcaceae*, *Erysipelotrichaceae*, and *Clostridiaceae*). Bifidobacteria use fermentation to produce SCFAs, mainly acetate and formate, during growth when carbohydrates are limited. Bifidobacteria alternatively produce acetate and lactate when carbohydrates are in excess [73]. Various dietary carbohydrates (called prebiotics) can selectively stimulate microbial growth and metabolic activity. A combination of probiotics and prebiotics (called a synbiotic) is powerfully able to shift the predominant bacteria and production of SCFAs. For examples, *L. rhamnosus GG* (LGG) with a mixture of prebiotics produces SCFAs. *Lactobacillus acidophilus* CRL 1014 was also recently shown to increase SCFAs (acetate/butyrate/propionate) when studied in a reactor called SHIME (Simulator of Human Microbial Ecosystem) [74]. Bifidobacteria such as *B. longum SP 07/03* and *B. bifidum MF 20/5* produce and release propionate and acetate, but not butyrate [75].

SCFAs may have beneficial effects on gut health through various mechanisms. SCFAs play an important role in maintaining metabolic homeostasis in colonocytes, and they protect colonocytes from external harm. SCFAs, especially butyrate, confer protection against the development of colorectal cancer (CRC) [76,77]. Butyrate promotes colon motility, reduces inflammation, induces apoptosis by inhibition of histone deacetylation, and inhibits tumor cell progression. Evidence points toward SCFA receptors in the colon, which includes both free fatty acid receptors (FFARs) and G-protein-coupled receptors (GPRs). FFAR3 (GPR41) and FFAR2 (GPR43) on colonocytes control motility [78]. SCFAs are able to bind and activate FFAR2 and/or FFAR3 located on intestinal epithelia, inducing glucagon-like protein-1 (GLP-1) and peptide tyrosine tyrosine (PYY) release into the basolateral milieu. Released GLP-1 and PYY activate enteric or primary afferent neurons in pelvic and vagal networks. Neural information travels to the central nervous system (CNS), affecting host metabolic energy expenditure [79]. SCFAs reduce neutrophil cytokine production [80], while reducing macrophage nuclear factor kappa-light-chain-enhancer of activated B cells (NF-κB) signaling [81], resulting in anti-inflammatory actions. Most importantly, butyrate has the ability to induce the differentiation of Tregs, which control intestinal inflammation [82]. However, the understanding of the underlying molecular mechanisms remains incomplete, mainly due to the lack of data on actual uptake fluxes of SCFAs under different conditions, i.e., with different dietary substrates, microbiota, and disease models. Most studies report concentrations of metabolites or transcript levels, but these do not necessarily reflect SCFA flux changes [75].

Under certain circumstances, treatment with probiotic lactobacilli could yield metabolites in the SCFA pathway that may be harmful. In the setting of short bowel syndrome, infants have been reported to develop D-lactic acidosis (which is not made by human cells) [83], because of the low abundance of the anaerobic microbiota capable of lactate utilization [84] and a dominance of lactobacilli in the feces [85].

*Tryptophan metabolism–aryl hydrocarbon receptor activation*. L-Tryptophan (Trp) plays crucial roles in the balance between intestinal immune tolerance and activation [86]. Recent studies have underscored the changes in the gut microbiota that modulate the host immune system by modulating Trp metabolism. Trp metabolites include host-derived Trp metabolites, such as kynurenines, serotonin, and melatonin, but also bacterially produced Trp metabolites, including indole, indolic acid, skatole, and tryptamine [87]. Trp metabolites are ligands of the aryl hydrocarbon receptor (AhR) [88]. Ahr is a cytosolic ligand-activated transcription factor in dendritic cells and T cells. AhR plays a critical role in maintaining gut immune tolerance and barrier function, as evidenced by the finding that AhR-null mice exhibit severe symptoms and mortality in animal models of dextran sodium sulfate (DSS)-induced colitis [89]. Ahr^−/−^ mice are more susceptible to intestinal challenge with toxins [90] and pathogens [91]. Studies have identified a critical mechanism of AhR in immune tolerance involving anti-inflammatory IL-22 production, which tolerizes intraepithelial T lymphocytes and innate lymphoid cells (ILCs) [92]. Host and bacterial Trp metabolites stimulate AhR and AhR-dependent gene expression, including IL-6, IL-22, prostaglandin G/H synthase 2 (PTGS2), vascular endothelial growth factor A (VEGFA), cytochrome P450 1A1 (CYP1A1), and mucin 2 (Muc2) in the intestine. These products individually and additively modulate intestinal homeostasis [87].

The effects of indolic acid derivatives produced from Trp by gut bacteria and probiotics have earned recognition as major metabolic products in this process. Metabolites such as indole-3-acetic acid (IAA), indole-3-aldehyde (IAId), indole acryloyl glycine (IAcrGly), indole lactic acid (ILA), indole acrylic acid (IAcrA), and indolyl propionic acid (IPA) all can impact intestinal homeostasis. For example, *Clostridium sporogenes* can convert Trp into IPA, which protects mice from DSS-induced colitis [93]. IPA significantly enhances anti-inflammatory cytokine IL-10 production after lipopolysaccharide (LPS) stimulation and reduces TNF-alpha production. The probiotic *Bifidobacteria infantis*, when given enterally, attenuates proinflammatory immune responses by elevating plasma Trp and kynurenic acid levels in rats [94]. The probiotic *Lactobacillus reuteri*, in the presence of luminal Trp, produces IAld, which is able to activate ILC3 cells to produce IL-22 via AhR, contributing to antifungal resistance and mucosal protection from inflammation [95]. In summary, as a therapeutic strategy, probiotic treatment in combination with Trp metabolism can alter the intestinal microbiota, increase the generation of AhR ligands, and ultimately protect the host from intestinal inflammation.

*TGF-β and Tregs*. Transforming growth factor-beta (TGF-β) is a multifunctional polypeptide with profound regulatory effects which affect many developmental and physiological processes. TGF-β in the intestinal mucosa is a key immunoregulatory molecule, shown to induce Tregs and to promote B-cell IgA production. One TGF-β signaling pathway activates the transcriptional factors SMAD2 and SMAD3 [96]. SMAD3 is a crucial transcription factor enhancing Foxp3 expression in Tregs. TGF-β induces Foxp3 gene transcription in thymic Treg precursors, and also converts naïve T cells into inducible Treg (iTregs), while protecting Tregs from apoptosis [97]. Probiotic bacteria have been shown to generate a Foxp3^+^ Treg response in the small intestine. Our study of experimental NEC models demonstrated that orally feeding *L. reuteri* 17938 increases the frequency of Foxp3^+^ Tregs in the intestinal mucosa to prevent the development of NEC [16,98]. *Lactobacillus gasseri SBT2055* induces TGF-β expression in dendritic cells and activates TLR2 signaling to produce IgA in the small intestine [99]. Probiotic VSL #3-induced TGF-β also ameliorates food allergy inflammation in a mouse model of peanut sensitization through the induction of Tregs in the gut mucosa [100]. The administration of *B. breve* to preterm infants also can upregulate TGF-β1 signaling and may possibly be beneficial in attenuating inflammatory and allergic reactions in infants [101]. In the setting of infectious enteritis, *L. acidophilus* attenuates *Salmonella typhimurium*-induced gut inflammation via TGF-β1 signaling [102].

*Nucleoside (adenosine) signaling*. We have identified a novel mechanism of *L. reuteri* 17938 in regulating multiorgan inflammation. *L. reuteri* modifies the microbiota–adenosine-inosine receptor 2A (A_2A_) axis, which in turn inhibits T_H_1 and T_H_2 cell differentiation to reduce inflammation in the liver, lungs, gut, and skin [103,104]. This mechanism was identified in the “scurfy” mouse model, in which genetic Treg deficiency induces autoimmune total body inflammation. Foxp3^+^ Treg cell deficiency in these mice results in gut microbial dysbiosis and autoimmunity over their entire lifespan. A severe autoimmune disease named IPEX syndrome (immunodysregulation, polyendocrinopathy, and enteropathy, with X-linked inheritance) is the parallel syndrome in humans [105]. Remodeling gut microbiota with *L. reuteri* 17938 markedly prolonged survival and reduced multiorgan inflammation in scurfy mice. We found that *L. reuteri* 17938 changed the metabolomic profile disrupted by Treg deficiency; and the predominant change was to restore serum levels of the purine metabolite inosine, alongside the downstream products xanthine and hypoxanthine. One of the key mechanisms of Tregs is to control inflammatory effector T cells (Tems). Tems include T_H_1, T_H_2, and T_H_17 subsets of T cells; these proinflammatory families of T cells are controlled via the interaction of adenosine (produced by Tregs) and the receptor A_2A_, which is highly expressed on T cells. In the absence of Tregs, the adenosine metabolite inosine at high doses may replace the effect of adenosine to interact with the A_2A_ receptor and inhibit T_H_ cell differentiation. When we fed inosine itself to Treg-deficient scurfy mice, we observed that inosine prolonged lifespan and inhibited multiorgan inflammation by reducing T_H_1/T_H_2 cells and their associated cytokines. Mechanically, the inhibition by *L. reuteri* and inosine of the differentiation of T_H_1 and T_H_2 cells depended on the A_2A_ receptor, which was confirmed by using an A_2A_ antagonist to block A_2A_ receptors [103] and by genetic knockout of the A_2A_ receptor in sf mice [104].

*Histamine signaling*. The tolerogenic effects of lactobacilli are very strain- and metabolite-dependent. For example, a *L. rhamnosus* strain that secretes low levels of histamine is immunosuppressive [106,107], whereas a *L. saerimneri* strain secreting high histamine levels induces gut inflammation [108]. In a series of studies, *L. reuteri* ATCC PTA 6475 (*L. reuteri* 6475) was found to differ from the sister strain *L. reuteri* 17938, in that *L. reuteri* 6475 makes histamine. Histamine is produced by *L. reuteri* 6475 via the action of histidine decarboxylase (HDC). Its production of histamine suppresses TNF-α synthesis in vitro [109]. Gao et al. showed that *L. reuteri* 6475 has anti-inflammatory effects in the trinitrobenzoate (TNBS) model of colitis via a mechanism dependent on intestinal histamine-2 receptor signaling [110]. A mutant *L. reuteri* 6475 strain lacking HDC did not suppress TNBS-induced colitis in mice; furthermore, the anti-inflammatory effect of *L. reuteri* 6475 was dependent on the histamine H-2 receptor on intestinal cells [111]. This HDC-dependent gene effect may be of relevance to colorectal carcinoma, which, interestingly, is more prevalent in humans deficient in HDC. As a proof of concept, *L. reuteri* 6475, when administered in a Hdc^−/−^ mouse model of colon cancer, suppressed tumor size and number, presumably by its synthesis of histamine [111].

## 4. “Polarization” within the Medical Community Regarding the Use of Probiotics

The medical community has not yet endorsed the use of probiotics. In fact, the U.S. Food and Drug Administration has not yet approved any probiotics for preventing or treating any health problem. Despite the numerous evidence-based reviews and meta-analyses cited herein, there are legitimate reasons for caution. Many of the meta-analyses suffer from the practice of lumping together different probiotics which may have widely different mechanisms of action. Some experts have warned that the rapid growth in the marketing of probiotics may have outpaced scientific research for many of their proposed uses and benefits [112]. More concerning is that there have been rare reports of bacteremia with cultures positive for the probiotic administered, leading to probiotic-associated endocarditis and even death. One notable case involved an infant who developed invasive mucormycosis, leading to intestinal perforation and death, resulting from a probiotic (ABD-Dophilus) which was contaminated with a fungus, *Rhizopus* *oryzae* [113]. However, overall, probiotic groups compared with matched placebo-treated controls have often shown a reduction in sepsis rates, as shown in preterm infants [114,115] and in adults following gastrointestinal surgery [116].

There are other concerns among skeptics.

*Numerical skepticism*. The argument is sometimes raised, “How can 1–100 billion colony-forming units (CFUs) of a probiotic outweigh the effects of 10–75 trillion commensals in the gut?”, noting a 1:1000 ratio of probiotic to commensal bacteria [117]. This numerical consideration is based on an assumption that a probiotic would need to establish itself (colonize) and differentiate in the large intestine. Consider the following: An infective dose of *E. coli* 0157:H7 of only 50 CFUs is sufficient to cause a potentially lethal bloody diarrhea in humans, leading to the hemolytic uremic syndrome [118]. It is actually remarkable that the previously mentioned body of research does show significant effects of probiotics in light of the sheer numbers of normal commensal microorganisms. However, the meta-analyses above show evidence of probiotic efficacy without significant colonic colonization. Most studies can show limited recovery of probiotics in the stool [119], but the number of colony-forming units (CFUs) for *L. reuteri* are on the order of 1:1000 of the dose administered and for *L. rhamnosus* GG are only 1:10,000 of the dose administered [120]. Another study showed fecal recovery of orally administered *L. fermentum* probiotic, but as in most studies, there was only a low level of the probiotic in the stool [121]. We have not consistently been able to identify by PCR significant numbers of probiotic in the stools—even while patients are actively on treatment [122,123]. Nevertheless, in our studies of *L. reuteri*, we have consistently found significant evidence of recognition by the host of the probiotic; for example, a mild elevation in the fecal level of the antimicrobial calprotectin (within the normal range) [124], a shift in microbial community composition, and an increase in circulating neutrophil count in infants with colic [125]. We believe a possible explanation lies in the observation that most lactobacilli and bifidobacilli are primarily small bowel colonizers, where they exert their immunologic effects.

*Publication bias*. It is generally recognized that clinical trials with negative findings are hard to publish. For this reason, meta-analyses will often contain a funnel plot, an asymmetry of which is a way of determining publication bias [126]. Funnel plots for probiotic studies have generally shown no publication bias for probiotics in most of the conditions described, such as NEC prevention [127], IBS improvement [128], *H. pylori* eradication [129], and amelioration of infant colic [130]. Nevertheless, there is much work to do in identifying optimal strains and using meta-analysis to prove the effect size of the disease (which in some cases may be significant, but only minor).

*Generalizability of findings*. Some have argued that a probiotic may be effective only in a well-defined, narrow population. For example, is there greater efficacy in children versus adults? Children less than 3–6 years old have an incompletely developed microbiome and may be more responsive to microbial manipulation. The strongest effect size for probiotics has been shown in pediatric studies; for example, the effect of probiotics in reducing the incidence of NEC (in the latest systematic reviews, the relative risk (RR) was 0.55, 95% confidence interval (CI) 0.43 to 0.70) or in shortening the course of acute infectious diarrhea (0.67 days, 95% CI –0.95 to –0.38) [131]. Another concern is whether probiotics may be more or less efficacious in different geographical locations, where populations have different dietary habits and differences in microbial exposure owing to differences in hygiene and food storage. This concern is reasonable, and broader meta-analyses including studies from different countries are indicated.

*Safety in immunodeficient states*. Finally, there is concern about giving chemotherapeutic agents or immunomodulators along with live microorganisms to patients who are immunocompromised. Children and adults with autoimmune diseases, such as lupus, ulcerative colitis, and rheumatoid arthritis, are often on immunosuppressive medications, biologics, or corticosteroids. Is it safe to give probiotics to these individuals? Our opinion is that it is safe and indicated. In fact, may the question may be better phrased, “Is it safer to give probiotics than not to withhold them?”, in view of the deleterious effects of patient exposure to multiple systemic antibiotics, resulting changes in microbiome, and alterations in barrier function of intestinal and other epithelial surfaces in these patients. Certainly, clinicians are quick to administer antimicrobial and/or antiviral agents to these individuals. There are numerous RCTs in the literature describing adults and children with cancer and immunodeficiency who have been treated with probiotics or placebo [132,133,134,135]. The most comprehensive review to date examined safety in immunocompromised adults using common terminology adverse event reporting. There were 57 studies in 4914 individuals, 2506 of whom received a probiotic or synbiotic. These included critically ill “intensive care unit” subjects, those with cancer, human immunodeficiency virus (HIV)-infected individuals, and those with arthritis, inflammatory bowel disease, or recent gastrointestinal surgery [136]. The authors concluded that probiotics were safe and, overall, associated with fewer adverse events compared to the control group. However, there were flaws in precise reporting in most of the cited studies. That report was in 2014, and it is likely that there will be upcoming reports and systematic reviews of probiotics in immunocompromised individuals.

## 5. The Future of Probiotics

Henri Poincare said in *The Foundations of Science* that, “It is far better to foresee even without certainty than not to foresee at all.”. Based on the collective evidence, the authors suggest the following events are likely to take place in the near future.

Probiotics are likely to be used in autoimmune diseases as a component of various treatment regimens. One size will not fit all. The choice of optimal probiotic or multispecies strains will evolve for each disease entity studied.

The present “third-party” insurance reimbursement problem will change. Currently, insurance plans in the U.S. cover antibiotics, but not probiotics; but (as discussed) a body of evidence is evolving to support that clinical outcomes will be improved with probiotics. Once safety issues in vulnerable populations are adequately addressed by properly controlled and regulated trials, we expect widespread use in children and adults with autoimmune disorders and (we hope for) coverage by insurance plans.

Quality improvement efforts by medical institutions will likely reward treatments with the best outcome. An example of this is the protocol for treatment of infants admitted to hospital with diarrheal dehydration at Cincinnati Children’s Hospital. An international working group selected care protocols for children with acute diarrhea, using systematic reviews, Delphi methodology, and external peer review. They decided that oral rehydration and probiotics were the only treatments recommended for infants presenting with acute diarrhea [137]. I nvestigators placed in the electronic order set an entry for the administration of *Lactobacillus rhamnosus* GG. After implementation of this initiative, the prescribing of this probiotic increased from 1% to 100% [138]. However, a retrospective study of 145 U.S. hospitals assessing ~1,900,000 hospital discharges showed that only in 2.6% of all hospitalizations were probiotics administered [139].

Novel delivery systems will facilitate probiotic delivery and efficacy. “Designer probiotics” is a term that has been given to probiotics with genetic engineering to facilitate delivery to the small intestine, enhance competitiveness within the gastrointestinal tract, and improve outcomes in certain disease states (reviewed in [140]). To overcome thermal and osmotic stress, probiotics have been suspended in high-osmolarity solutes such as betaine. Additionally, expression cloning of solute-uptake genes for the betaine transporter BetL by *Bifidobacterium breve* resulted in higher fecal levels of the probiotic in murine stools, probably because of improved survival in the hyperosmotic upper small intestinal lumen. Recently, an *E. coli* strain was engineered to secrete HIV gp41–hemolysin A hybrid peptides. These peptides block HIV entry into target cells. There are two other studies demonstrating the potential use of designer probiotics in protecting from HIV infection [140]. Another interesting way to magnify probiotic retention and clinical impact is to administer the organism with agents that promote biofilm formation. Recently, Olsen et al. administered *L. reuteri* grown as a biofilm on the surface of dextranomer microspheres (DM) loaded with mannitol and sucrose. A single dose administered to newborn rat pups was sufficient to reduce the severity of necrotizing enterocolitis [141].

Probiotic products may in some cases replace the probiotics themselves. Metabolites may be identified that can be given instead of or along with live microorganisms. Mechanistic studies have begun to unravel the secrets of probiotic effects. Metabolites mentioned above, including short-chain fatty acids, growth factors, bacteriocins, tryptophan metabolites, and adenosine derivatives, could be beneficial. If the optimal, most potent metabolite were identified for a given disease, it may be possible to achieve the probiotic effect without the inherent risks of live cultures. However, it is possible that sustained luminal levels may not be attained with such an approach or that the effect of the probiotic requires the synthesis of metabolites by microbial consortia.

Finally, the scientific community may begin to refer to probiotics as evidence-based, rather than “alternative” medicine.
